# Regional mortality by socioeconomic factors in Slovakia: a comparison of 15 years of changes

**DOI:** 10.1186/s12939-016-0404-y

**Published:** 2016-07-19

**Authors:** Katarina Rosicova, Lucia Bosakova, Andrea Madarasova Geckova, Martin Rosic, Marek Andrejkovic, Ivan Žežula, Johan W. Groothoff, Jitse P. van Dijk

**Affiliations:** Kosice Self-governing Region, Department of Regional Development, Land-use Planning and Environment, Nam. Maratonu mieru 1, 042 66 Kosice, Slovakia; Graduate School Kosice Institute for Society and Health, Pavol Jozef Safarik University, Kosice, Slovakia; Department of Health Psychology, Faculty of Medicine, Pavol Jozef Safarik University, Kosice, Slovakia; Faculty of Business Economics, Department of Quantitative Methods, University of Economics in Bratislava, Kosice, Slovakia; Olomouc University Society and Health Institute, Palacky University Olomouc, Olomouc, Czech Republic; Faculty of Humanities and Natural Sciences, University of Presov, Presov, Slovakia; Institute of Mathematical Sciences, Faculty of Science, Pavol Jozef Safarik University, Kosice, Slovakia; Department of Community and Occupational Health, University Medical Center Groningen, University of Groningen, Groningen, The Netherlands

**Keywords:** Income, Material needs, Mortality, Regional differences, Unemployment

## Abstract

**Background:**

Like most Central European countries Slovakia has experienced a period of socioeconomic changes and at the same time a decline in the mortality rate. Therefore, the aim is to study socioeconomic factors that changed over time and simultaneously contributed to regional differences in mortality.

**Methods:**

The associations between selected socioeconomic indicators and the standardised mortality rate in the population aged 20–64 years in the districts of the Slovak Republic in the periods 1997–1998 and 2012–2013 were analysed using linear regression models.

**Results:**

A higher proportion of inhabitants in material need, and among males also lower income, significantly contributed to higher standardised mortality in both periods. The unemployment rate did not contribute to this prediction. Between the two periods no significant changes in regional mortality differences by the selected socioeconomic factors were found.

**Conclusions:**

Despite the fact that economic growth combined with investments of European structural funds contributed to the improvement of the socioeconomic situation in many districts of Slovakia, there are still districts which remain “poor” and which maintain regional mortality differences.

## Background

Most Central and Eastern European countries have passed through a period of turbulent changes affecting the socio-political context which sets the social determinants of health [1]. Some of the EU-member states which entered in 2004 or later, including Slovakia, have experienced a period of economic growth and huge investments of European structural funds. As in most European countries, mortality in Slovakia has shown a declining tendency since the turning point of 1989 [2]. Significant differences exist on the regional level [3], even though some areas have not improved or have performed even worse. Therefore, it is of interest to study factors that change over time and simultaneously contribute to the standardised mortality rate (SMR) in order to explain differences in the changes in mortality over time.

The associations of income, unemployment and poverty with mortality are seen as very important and have been previously discussed [4–8]. Income is one of the main determinants influencing not only survival but also death [8]. Mortality decreases significantly when income increases [9], although the opposite effect has also been suggested [10]. The most frequent income-mortality associations are usually studied at a single point in time [5], though a few papers have investigated this relation as a trend over time [11, 12], both confirming [12] and contradicting the effect of time [11].

Unemployed persons have a higher risk of premature death than those who are employed [7]. A time trend study performed in the United States at the national level confirmed that increased unemployment was associated with a substantial increase in mortality [13]. A Swedish time trend study presented contrasting findings showing a correlation between higher unemployment and lower mortality [14], while another regional study did not find any relation between unemployment and overall mortality [15].

Poverty is also associated with mortality [4]. The convention is to measure poverty in terms of absolute income [16]. However, this has met with criticism, so also an increasing number of measures based on the construct of ‘material deprivation’ [16] is used. Material deprivation refers to the inability of individuals or households to afford consumer goods and activities that are typical in a society [17]. Benefits regarding material need can be considered as a solid poverty indicator, as such benefits are set to fill the gap between the person’s income and his needs, and anyone whose income is below a minimum level is entitled to receive them [18]. However, studies on the association between poverty defined as the recipients of material need benefits and mortality are scarce [19, 20]. Andrén and Gustafsson [19] showed that benefit recipients have twice the probability of death as non-recipients. Naper [20] found that the all-cause mortality of benefit recipients in Norway was considerably higher than that of the general population.

Socioeconomic differences in mortality seem to be gender-specific, although findings contrast with one another [7, 10]. Regarding the association between income and mortality, some studies have shown that the results were the same for both sexes [21, 22]. On the other hand, Fukuda et al. [10] showed a significant association between higher income and higher mortality in females only and a stronger unemployment-mortality relationship in males. In Slovakia, socioeconomic indicators in regional mortality were found only among males, where unemployment significantly contributed to mortality differences but income did not [23, 24].

Findings on associations between income and regional mortality are scarce, while those between unemployment and regional mortality are contradictory and between recipients of material need benefits and mortality are limited. The pattern between these indicators and mortality seems to differ by gender. Furthermore, Slovakia passed through a period of huge societal changes which might influence regional differences in both socioeconomic indicators and mortality. The aim of the study was to analyse the associations between selected socioeconomic indicators (unemployment, income, recipients of material need benefits) and the SMR in the population aged 20–64 years by gender in the districts of the Slovak Republic in the periods 1997–1998 and 2012–2013.

## Methods

### Study population

The study population covers all of the inhabitants of the Slovak Republic aged 20–64 years in two periods: 1997–1998 and 2012–2013. The selected age group is primarily the economically active population integrated into the labour market. This part of the population has the relatively lowest mortality rate by age, has finished the process of education and receives a certain kind of income, either as a salary or as social security benefits.

The average number of inhabitants aged 20–64 years in the Slovak Republic as of July 1^st^ during the period 1997–1998 was 3,185,682 people (49.4 % men); in the period 2012–2013 it was 3,551,193 people (50.0 % men). The total number of deaths among those aged 20–64 years over the two-year period of 1997–1998 was 29,239 (70.8 % men), and in 2012–2013 was 28,817 (71.3 % men). Thus, on average about 14 thousand deaths occurred each year.

To be able to study regional differences, the study population was analysed at the district level using an ecological study design. The Slovak Republic is divided into 8 regions at the regional level NUTS 3 (Nomenclature of Territorial Units for Statistics) and further into 79 districts at the local level LAU 1 (Local Administrative Units), 5 of which constitute the capital city Bratislava and 4 the second largest city, Košice. The average number of inhabitants aged 20–64 per district in the period 1997–1998 was 40,321 persons, ranging from 7240 to 97,297 inhabitants, and in the period 2012–2013 this was 44,952 persons, ranging from 7668 to 108,967.

### Data

The data consist of absolute population numbers and numbers of deaths by gender in the districts of the Slovak Republic in the periods 1997–1998 and 2012–2013 and were obtained from the Statistical Office of the Slovak Republic [25].

The unemployment rate, income and the proportion of inhabitants in material need in a district were used as economic indicators associated with the mortality rate. All indicators were calculated for each district in the two separate periods of 1997–1998 and 2012–2013.

The unemployment rate was expressed as the proportion of the number of unemployed inhabitants aged 20–64 years to the total number of economically active population by gender. Numbers of unemployed and economically active population by gender were obtained from the tally of the Centre of Labour, Social Affairs and Family of the Slovak Republic [26]. The income level (average monthly gross income) was based on data from the Statistical Office of the Slovak Republic. At the district level income data are available only in the form of gross income for companies with 20 or more employees (about 60 % of all companies in the country) [27, 28]. The numbers of recipients of benefits in material need were obtained from the tally of the Centre of Labour, Social Affairs and Family of the Slovak Republic [29] and included all persons in a household whose combined household income is below the subsistence minimum level annually established by law, this being 194.58€ in 2012. The percentage of the inhabitants in material need is expressed as the proportion of the total number of recipients of benefits in material need to the total number of population.

**Table 1:** Basic data for the Slovak population aged 20–64 years – averages for the periods 1997–1998 and 2012–2013

	1997–1998			2012–2013		
	Males	Females	Total	Males	Females	Total
Standardised mortality (per 100,000 inh.)	658.0	264.9	459.1	578.1	233.0	405.7
Unemployment rate	11.8 %	13.1 %	12.4 %	13.6 %	15.8 %	14.6 %
Income level	251€^a^	188€^a^	221€^a^	1001€	761€	886€
% in material need			3.6 %			3.4 %

^a^recalculated by average annual rate of the EUR at the end of 1999

Source: Data from the Centre of Labour, Social Affairs and Family of the Slovak Republic and from the Statistical Office of the Slovak Republic

### Measures of mortality

Using the regional mortality data, the SMR was calculated. For each region the mortality by 5-year age-groups (20–24, 25–29, 30–34, 35–39, 40–44, 45–49, 50–54, 55–59, 60–64) and the total mortality rate by gender were calculated. Regional mortality rates were standardised by the direct method of standardisation and by age using the Slovak population as the standard. The mortality rate is expressed as the number of deaths per 100,000 inhabitants.

### Statistical analysis

Linear regressions were applied: regional differences in SMR were set as the dependent variable; the unemployment rate, income and the proportion of those in material need were set as independent variables. Initially the crude effect of each factor was analysed separately and then all factors were included into the final model. Both analyses were done separately for both periods and for males and females. The regression models were checked for collinearity. To check changes in the coefficients between two periods the F-test was used.

Analyses were done using SPSS version 20.0.

### Maps

Maps were constructed using regional SMR and data by socioeconomic indicators. The range of the indicators on the maps was divided into quartiles.

Maps were created using ArcView.

## Results

### Mortality

**Fig. 1:**
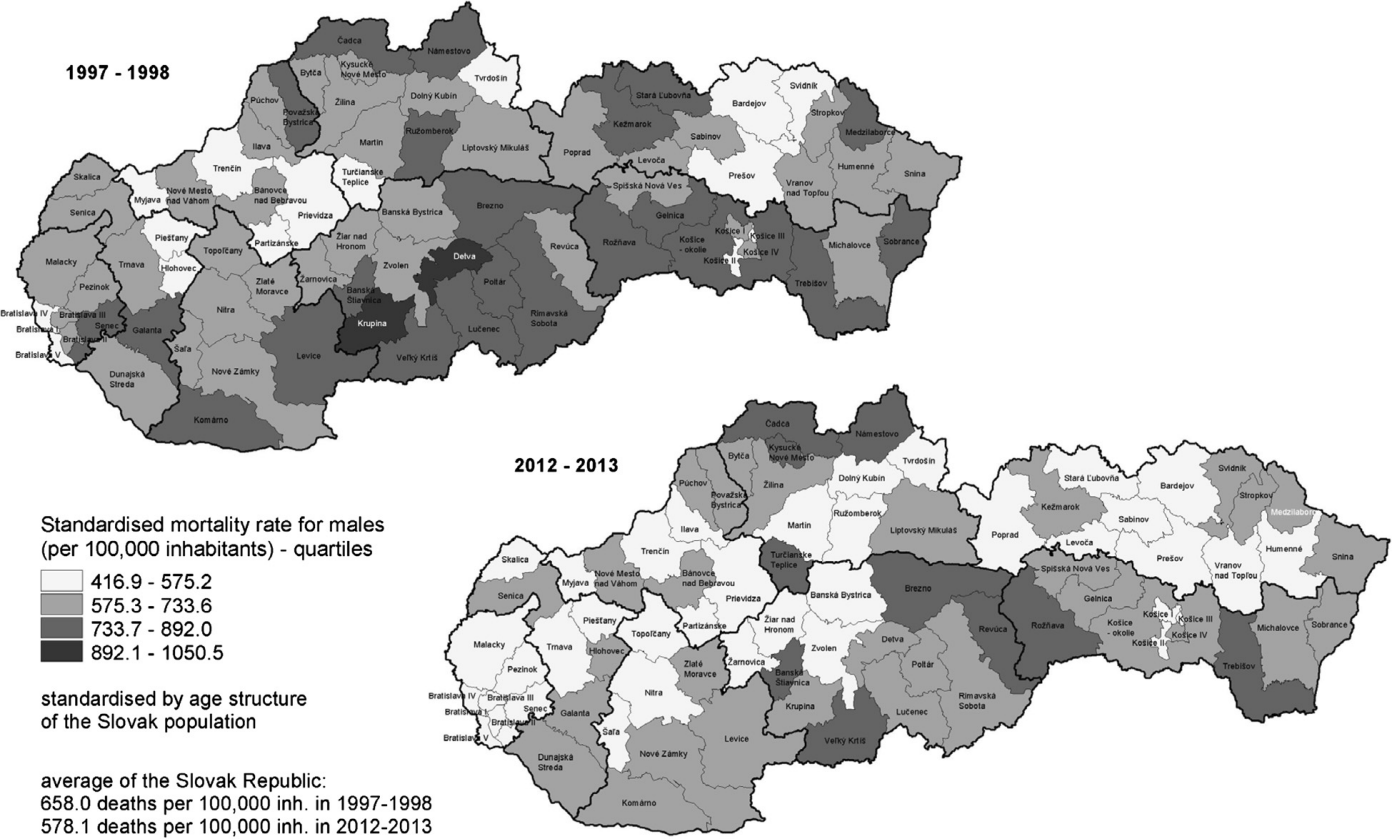
Standardised mortality rates for males aged 20–64 years by districts in the Slovak Republic in the periods 1997–1998 and 2012–2013. Source: Data from the Statistical Office of the Slovak Republic

The SMR for males aged 20–64 years in the districts ranged from 444.9 to 1050.5 deaths per 100,000 inhabitants in the period 1997–1998 and from 416.9 to 850.1 deaths per 100,000 inhabitants in the period 2012–2013, pointing to a reduction in the range of mortality rates over the period 2012–2013. Compared with the mortality rate for males aged 20–64 years at the national level, in both periods half of the districts (41 in the period 1997–1998 and 38 in the period 2012–2013 out of 79) achieved a lower SMR than the average rate for the Slovak Republic (Table 1, Fig. 1). Between the periods 1997–1998 and 2012–2013 the SMR among the male population declined in the majority of the districts (66 of 79; 84 %).

**Fig. 2:**
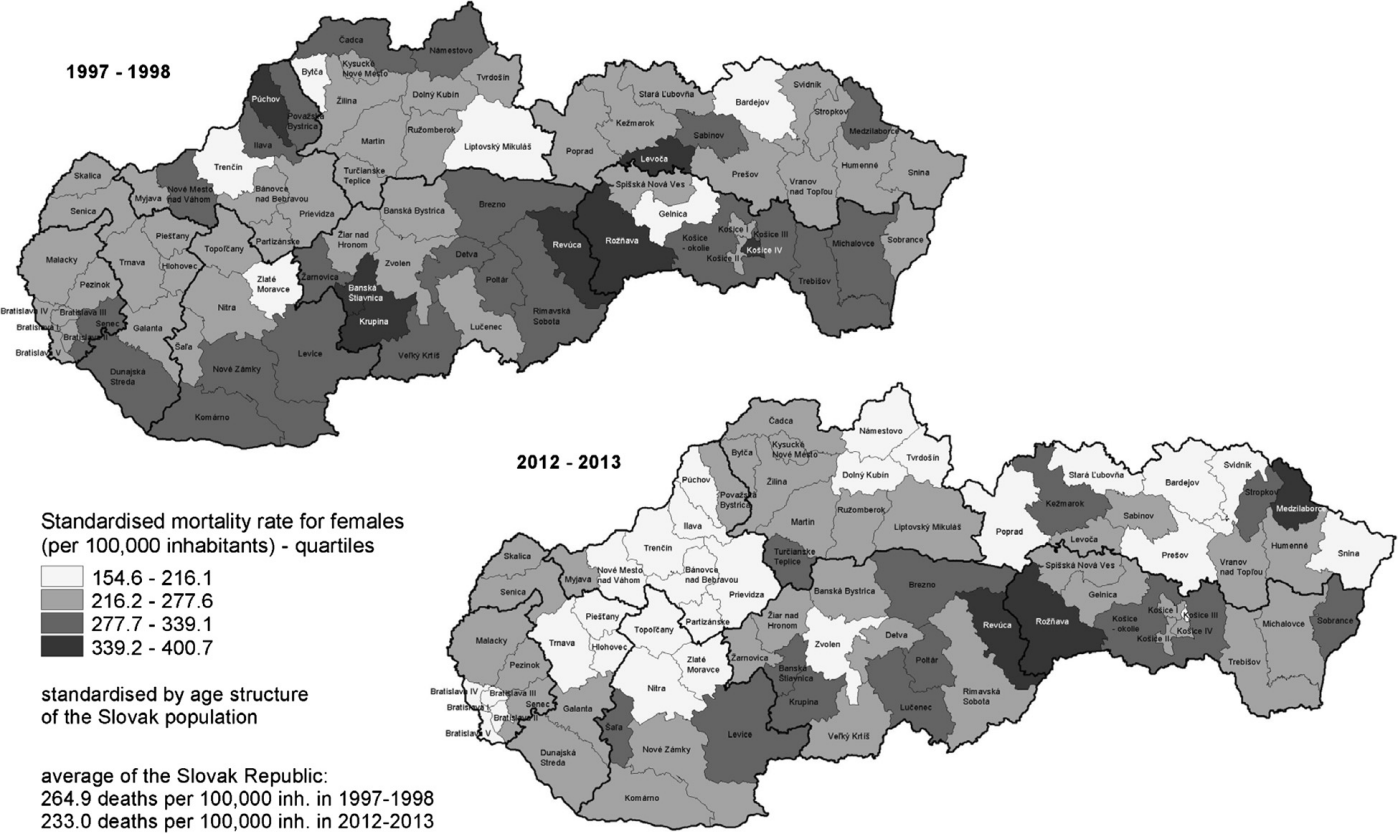
Standardised mortality rates for females aged 20–64 years by districts in the Slovak Republic in the periods 1997–1998 and 2012–2013. Source: Data from the Statistical Office of the Slovak Republic

The SMR for females aged 20–64 years in the districts ranged from 175.0 to 400.7 deaths per 100,000 inhabitants in the period 1997–1998 and from 154.6 to 391.9 deaths per 100,000 inhabitants in the period 2012–2013, and half of the districts (41 in the period 1997–1998 and 39 in the period 2012–2013 out of 79) attained a lower SMR for females aged 20–64 years than the average national mortality rate. Also, in the female population aged 20–64 years the SMR declined in the majority of districts (62 from 79; 78 %) and the range of the mortality rates in the period 2012–2013 slightly increased (Fig. 2).

**Fig. 3:**
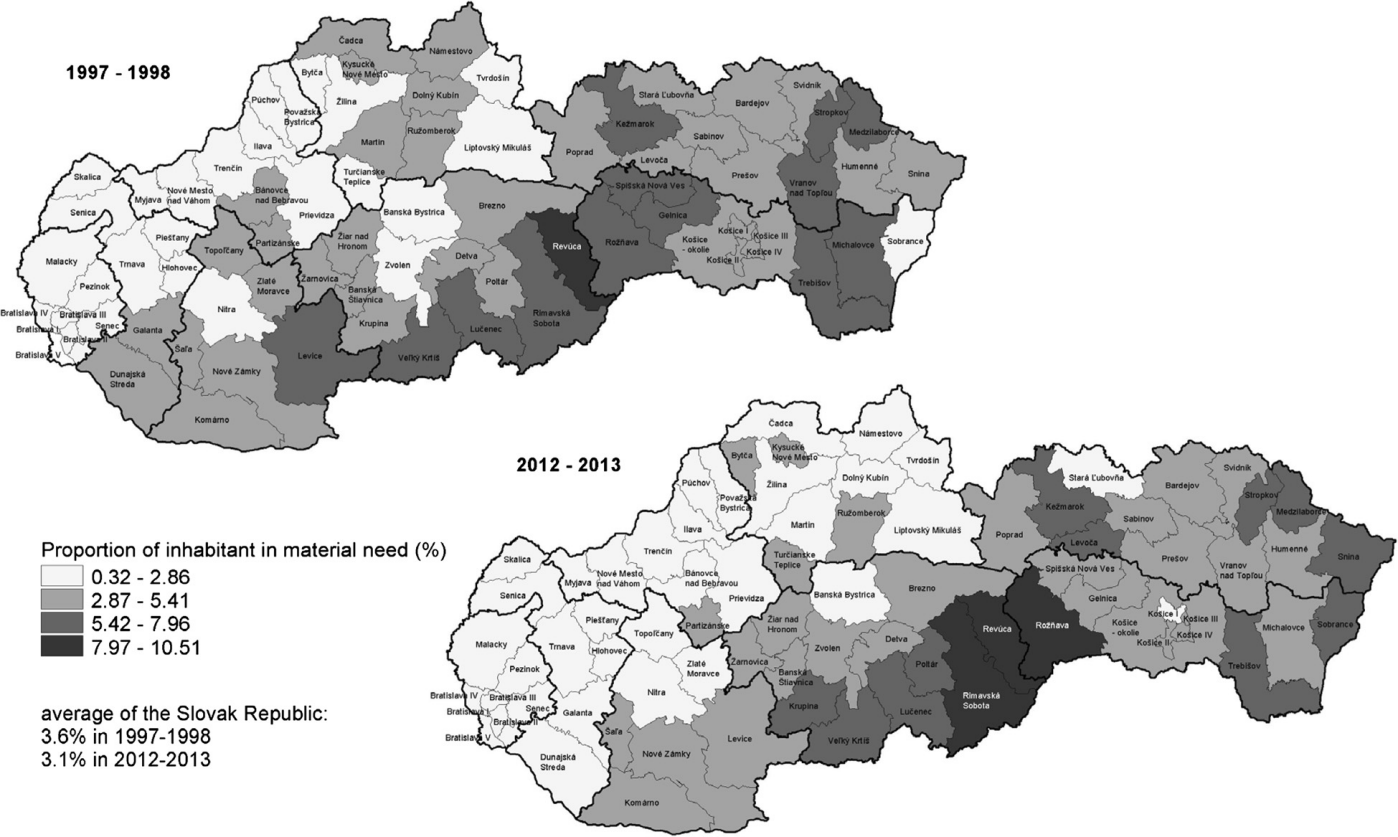
Proportion of inhabitants in material need by districts in the Slovak Republic in the periods 1997–1998 and 2012–2013. Source: Data from the Centre of Labour, Social Affairs and Family of the Slovak Republic

The proportion of inhabitants in material need in the districts ranged from 0.3 to 9.3 % in the period 1997–1998 and from 0.5 to 10.5 % in the period 2012–2013, pointing to a small increase in the proportion of inhabitants in material need over the period 2012–2013 (Fig. 3). Between the periods 1997–1998 and 2012–2013 the percentage of inhabitants in material need declined in the majority of districts (42 from 79; 53 %), but increased in one third of them. Compared with the percentage of the inhabitants in material need at the national level, in both periods more than half of the districts (43 in the period 1997–1998 and 46 in the period 2012–2013 out of 79) achieved a lower proportion of inhabitants in material need than the average for the Slovak Republic (Fig. 3).

### Linear regression

Table 2 presents the results of linear regression of the SMR in the districts of the Slovak Republic and separate economic indicators in the periods 1997–1998 and 2012–2013. In this model the variables were entered consecutively in order to explore the effects separately. The dependent variable is the SMR by district separately for males and females. Among males, in both periods, all selected socioeconomic indicators were significantly associated with the standardised mortality. Among females, in both periods, the unemployment rate and the proportion of inhabitants in material need were significantly associated with the standardised mortality, and income did not contribute to the prediction of the SMR in the period 1997–1998. In the period 2012–2013 an increase in the explained variance of all significant indicators in both genders could be observed.

**Table 2:** Linear regression between standardised mortality rates of those aged 20–64 years and economic indicators (separately) for the periods 1997–1998 and 2012–2013

Economic indicators (separately)	Male			Female		
	Standardised Coefficients (Beta)	Sig.	R^2^	Standardised Coefficients (Beta)	Sig.	R^2^
1997–1998						
Unemployment rate	.447	.000***	.200	.244	.030*	.059
Income	-.489	.000***	.240	-.130	.254	.017
Proportion of inhabitants in material need	.469	.000***	.220	.321	.004**	.103
2012–2013						
Unemployment rate	.431	.000***	.186	.570	.000***	.325
Income	-.380	.001**	.144	-.260	.020*	.068
Proportion of inhabitants in material need	.497	.000***	.247	.701	.000***	.491

*p ≤ 0.05; **p ≤ 0.01; ***p ≤ 0.001 (2-tailed); R^2^ – explained variance

Source: Data from the Centre of Labour, Social Affairs and Family of the Slovak Republic and from the Statistical Office of the Slovak Republic

The relationship between the SMR for inhabitants aged 20–64 years by gender and economic indicators together in the districts of the Slovak Republic in the periods 1997–1998 and 2012–2013, as revealed by linear regression, is presented in Table 3. The model explores the associations of all variables together with the mortality rates. The dependent variable is the SMR by district separately for males and females (continuous); the independent variables are selected economic indicators by district separately for males and females (all continuous). The model explained 28.1 % of the variance in SMR among the districts for males, 8.2 % of the variance in SMR among the districts for females in the period 1997–1998, and 25.5 % for males and 50.5 % for females in the period 2012–2013. The adjusted regression model shows that the proportion of inhabitants in material need significantly contributed to the prediction of the SMR for both genders in the districts of Slovakia in both periods. Among males income was also associated with a higher SMR in the districts of the Slovakia in period 1997–1998. The lower the income and the higher the proportion of inhabitants in material need, the higher the SMR was.

**Table 3:** Linear regression between standardised mortality rates of those aged 20–64 years and economic indicators (together) for the periods 1997–1998 and 2012–2013

Economic indicators (together)	Male		Female	
	Standardised Coefficients (Beta)	Sig.	Standardised Coefficients (Beta)	Sig.
1997–1998				
Unemployment rate	-.243	.344	-.308	.307
Income	-.394	.003**	-.017	.905
Proportion of inhabitants in material need	.480	.039*	.593	.031*
R^2^/Adjusted R^2^	.309 / .281		.117 / .082	
2012–2013				
Unemployment rate	-.418	.147	-.344	.130
Income	-.225	.084	.076	.484
Proportion of inhabitants in material need	.760	.005*	1.055	.000***
R^2^/Adjusted R^2^	.283 / .255		.524 / .505	

*p ≤ 0.05; **p ≤ 0.01; R^2^ – explained variance

Source: Data from the Centre of Labour, Social Affairs and Family of the Slovak Republic and from the Statistical Office of the Slovak Republic

Collinearity and its influence on the model were also tested. The results of collinearity show that the model did not appear to have a substantial problem (maximum condition number 29.8).

The F-test was used to check changes in the coefficients between the two periods. The regression coefficients were found to be identical in females (p-value 5.8 %). In males we found a significant change in the constant, while all other regression coefficients are the same (*p*-value 55.1 %).

## Discussion

We analysed the associations between selected socioeconomic indicators and SMR in the population aged 20–64 years by gender in the districts of the Slovak Republic in the periods of 1997–1998 and 2012–2013. In the Slovak population excess male mortality was observed in both selected periods, which is a typical phenomenon, however, for the structure of deaths by gender and age in the population of developed countries. Selected indicators were examined in relation to SMR, first separately and then together in the mutual model. A higher proportion of inhabitants in material need, and among males also lower income were significantly associated with higher SMR in both periods. The unemployment rate did not contribute to this prediction in either gender or in either period. We further found in the studied periods an increase of the explained variance of the SMR in females. Despite changes in the analysed variables and their distribution over the districts in the studied periods, we did not show significant changes in regional (i.e. district) mortality differences by the selected socioeconomic factors.

In most studies examining the association between income and mortality, as in our study, a negative relationship was found [11, 12]. It seems that higher income can be used to purchase healthier food, to invest in better health care, housing, schooling and recreation [30], which might have a distinct effect on mortality [31]. In our study a significant relation was confirmed only among men, which is different from other studies [21, 22]. Income may be a better indicator of men’s material conditions, as their incomes generally comprise the greater part of a household’s purchasing power. It may be also more important for self-esteem and self-respect among men, as work stands for a greater part of their life and they are considered to be the “bread-winners” [32].

Our findings on the association between unemployment and SMR in districts were not consistent, which is in accordance with study of Svensson [15]. This is in contrast to Brenner [13], who shows a clear relationship between the unemployment rate and mortality. The lack of an association between the unemployment rate and SMR in Slovak districts in the adjusted regression model might be caused by the fact that unemployed people in Slovakia are at the same time the recipients of material need benefits, which might mask the contribution of unemployment. However, we did not find indications for multicollinearity.

The high mortality rate among recipients of material need benefits may be determined by a general susceptibility leading to higher mortality from all causes, so there is an overall health risk associated with being a benefits recipient [20]. Moreover, poverty is closely linked to a variety of behaviours that impact mortality (smoking, alcohol abuse, physical inactivity, etc.) [20] and above all, benefits recipients seem to be more frequently exposed to violence than others [19]. Consequently, it seems that receiving a social benefit does not serve as a (sufficient) protective factor with regard to health as intended, as it is a risk factor regarding regional differences in SMR.

Despite the substantial economic growth between the periods 1997–1998 and 2012–2013 combined with the investments from European structural funds to the Central European countries, there are regions which remained “untouched” and “unimproved” in terms of poverty expressed in the proportion of inhabitants in material need, which significantly contributed to the regional differences in SMR. Moreover, there is a high probability that similar findings might also be found in other Central European countries.

### Strengths and limitations of the study

The strength of our study is the combination of the area-based design, age specification of the population and the over-time perspective. An ecological study uses data that generally already exist and is a quick and cost-efficient approach compared with individual level studies. It is also particularly valuable when an individual level association is evident and an ecological level association is assessed to determine its public health impact. The deficient databases regarding income (average monthly gross payment), which is available only for companies with 20 or more employees at the district level in Slovakia, is one of the main limitations of our study. The proportion of such enterprises is about 60 %, although the total number of enterprises cannot be determined due to the lack of available data. It would be very interesting to identify income as a variable which also includes data from small enterprises and to use it in a similar analysis on mortality rate, as is done in this paper.

### Implications

The results of our study indicate that a higher proportion of inhabitants in material need, and among males also lower income significantly contributed to higher standardised mortality in both periods. The main contribution of this paper is focusing of attention on decreasing the proportion of inhabitants in material need in Slovakia with the aim of reducing mortality rates. Our findings can be used in the development of social policies which should preferably increase employment in the regions with the highest proportion of inhabitants in material need, since probably only surveys, suitable policies and interventions will be able to “revitalise” regions at risk. Such a redistribution of parts of the government over the country could contribute to the reduction of the proportion of inhabitants in material need and thus reduce the SMR in such regions. In particular, these are the districts of the southern and eastern regions of the Slovak Republic, which had most of the unemployed, infrastructure lag and also deformed age structure reflecting unfavourable population development [33].

Factors that were analysed in this paper still create a prerequisite for further exploration of this field, where in addition to examining the overall level of income it would be appropriate to consider income inequality within a region and through the Gini coefficient, similarly as in studies carried out previously [34, 35].

## Conclusion

In conclusion, the proportion of inhabitants in material need, and among males also lower income seems to be the strongest predictors of standardised mortality in the population aged 20–64 years in the districts of the Slovak Republic in the periods 1997–1998 and 2012–2013. Despite the fact that economic growth combined with investments of European structural funds contributed to the improvement of the socioeconomic situation in many districts of Slovakia, there are still districts which remained “poor”, what maintained the regional mortality differences.

## Abbreviations

SMR, standardised mortality rate; NUTS, Nomenclature of Territorial Units for Statistics; LAU, Local Administrative Units
